# Inulin-Type β2-1 Fructans have Some Effect on the Antibody Response to Seasonal Influenza Vaccination in Healthy Middle-Aged Humans

**DOI:** 10.3389/fimmu.2015.00490

**Published:** 2015-09-22

**Authors:** Amy R. Lomax, Lydia V. Y. Cheung, Paul S. Noakes, Elizabeth A. Miles, Philip C. Calder

**Affiliations:** ^1^Human Development and Health Academic Unit, Faculty of Medicine, Southampton General Hospital, University of Southampton, Southampton, UK; ^2^National Institute for Health Research Southampton Biomedical Research Centre, Southampton General Hospital, University Hospital Southampton NHS Foundation Trust and University of Southampton, Southampton, UK; ^3^Department of Biological Sciences, Faculty of Science, King Abdulaziz University, Jeddah, Saudi Arabia

**Keywords:** prebiotic, oligofructose, immune response, vaccination, antibodies

## Abstract

β2-1 fructans are prebiotics and, as such, may modulate some aspects of immune function. Improved immune function could enhance the host’s ability to respond to infections. There is limited information on the effects of β2-1 fructans on immune responses in humans. The objective of the study was to determine the effect of a specific combination of long-chain inulin and oligofructose (Orafti^®^ Synergy1) on immune function in middle-aged humans, with the primary outcome being response to seasonal influenza vaccination. Healthy middle-aged humans (45–63 years of age) were randomly allocated to consume β2-1 fructans in the form of Orafti^®^ Synergy1 (8 g/day; *n* = 22) or maltodextrin as control (8 g/day; *n* = 21) for 8 weeks. After 4 weeks, participants received the 2008/2009 seasonal influenza vaccine. Blood and saliva samples were collected prior to vaccination and 2 and 4 weeks after vaccination. They were used to measure various immune parameters. The primary outcome was the serum concentration of anti-vaccine antibodies. Serum antibody titers against the vaccine and vaccine-specific immunoglobulin concentrations increased post-vaccination. Antibodies to the H3N2-like hemagglutinin type 3, neuraminidase type 2-like strain were higher in the Synergy1 group (*P* = 0.020 for overall effect of treatment group), as was serum vaccine-specific IgG1 2 weeks post-vaccination (*P* = 0.028 versus control). There were no other differences between groups in antibody titers or anti-vaccine immunoglobulin concentrations, in blood immune cell phenotypes, or in a range of immune parameters. It is concluded that Orafti^®^ Synergy1, a combination of β2-1 fructans, can enhance some aspects of the immune response in healthy middle-aged adults, but that this is not a global effect.

## Introduction

The prebiotic effect is defined as the selective stimulation of the growth and/or activity of gut microbes that confer health benefits to the host (1), as discussed elsewhere (2). β2-1 fructans are considered to be prebiotics. Inulin is a naturally occurring β2-1 fructan. Inulin can vary in chain length and can be hydrolyzed to shorter chain length oligofructose molecules. Orafti^®^ Synergy1 (referred to hereafter as Synergy1) contains a 50:50 (w/w) mixture of long-chain inulin and oligofructose. β2-1 fructans have been reported to modulate the intestinal microbiota (3–9), specifically increasing the numbers of bifidobacteria and lactobacilli. It is thought that bifidobacteria and lactobacilli influence the host immune system, improving its function (10). So far, there is no consensus on the best method to assess immune function in human studies (11–15). Vaccination is a controlled way to expose the immune system to a specific amount and type of antigen. It has been proposed that the body’s response to this antigenic exposure is the most relevant way in which to assess the functioning of the immune system, in the absence of an infectious challenge (12–15).

Several studies have been performed using response to vaccination as a tool to examine the effect of β2-1 fructans upon human immune function (16–26); although in most cases, the β2-1 fructans were used in combination with other potentially bioactive ingredients. Of these 11 studies, 4 found an effect of β2-1 fructans upon the vaccine-specific response (16, 17, 19, 20), while 7 did not (18, 21–26). These studies were performed in infants or children (16, 18, 21, 23–26) or in elderly adults (17, 19, 20, 22). Of the four studies carried out in the elderly, only one used β2-1 fructans alone (17), the remaining studies used supplements also containing vitamins, minerals, fats, or probiotics (19, 20, 22). Thus, there are few studies of β2-1 fructans alone on the immune response to vaccination in adult humans and there are no studies in middle-aged humans who may be considered to be a target group of consumers.

The aim of this study was to evaluate the effect of Synergy1 (8 g/day) on immune function in middle-aged humans using a double-blind randomized controlled trial design with the primary outcome being response to seasonal influenza vaccination determined as serum anti-vaccine antibody concentrations. We have previously reported that 4 weeks supplementation with Synergy1 (8 g/day) increased total and relative numbers of bifidobacteria in feces, but that there was no effect on a range of immune parameters measured in the absence of an *in vivo* immune challenge (9). We hypothesized that Synergy1 will enhance the serum antibody response to seasonal influenza vaccination.

## Participants, Materials, and Methods

### Participants

Participants (*n* = 49) were recruited via posters, word of mouth, e-mail, and newspaper/magazine advertisements. Both men and women were recruited. The inclusion criteria were as follows: age between 45 and 65 years; body mass index (BMI) between 20 and 32 kg/m^2^; not consuming prebiotic or probiotic supplements, drinks, or foods; in general good health; no antibiotic use in the 2 months prior to entering the study or during the study; and not having been vaccinated with the 2008/2009 seasonal influenza vaccine. The exclusion criteria were as follows: being type 1 or type 2 diabetic; displaying manifestations of allergy (asthma, hay-fever, or dermatitis) or being treated for these; being egg allergic; use of any prescribed medication (unless deemed acceptable by the principle investigator); suffering from any infectious illness or chronic gastrointestinal problem (e.g., irritable bowel syndrome, inflammatory bowel disease, cancer); recent blood donation; participation in another clinical trial; consuming vitamin, fish oil, evening primrose oil, or mineral supplements. The 2008/2009 vaccine included an A/Brisbane/59/2007 (H1N1)-like, an A/Brisbane/10/2007 (H3N2)-like, and a B/Florida/4/2006 (B)-like virus strain. Although seasonal influenza vaccines always include H1N1-, H3N2- and B-like virus strains, the specific strains used in the 2008/2009 vaccine had not been used previously (27). The Southampton and South West Hampshire Local Research Ethics Committee approved the study (09/H0504/2) and clinical governance was provided by Southampton University Hospitals NHS Trust Research and Development. All participants provided written informed consent. The study was registered at www.clinicaltrials.gov as NCT00898599.

### Study design and intervention

The study was a randomized, double-blind, controlled trial, with an 8-week intervention period; only data from the final 4 weeks are reported here. Data from the first 4 weeks of the intervention (i.e., prior to vaccination) have been reported elsewhere, and there was no effect on any immune parameter measured, despite an increase in the number of bifidobacteria in feces (9). Allocation to group [Synergy1 (*n* = 25); control (maltodextrin; *n* = 24)] was done by random selection of a sealed envelope containing a treatment code. Unblinding did not occur until after completion of all statistical analyses. Following randomization, participants underwent a 2-week run-in period, during which they limited their consumption of prebiotic and probiotic containing foods, and these restrictions continued throughout the study. Upon completion of this run-in, participants began the 8-week period of supplementation. Synergy1 and maltodextrin were provided as powders within coded, sealed paper sachets (4 g/sachet) by Beneo-Orafti, and were identical in appearance and packaging, except for the labeling (A or B). Participants were asked to consume two sachets per day (one in the morning and one in the evening yielding a total daily intake of 8 g) by stirring the contents into a glass of water. Participants were given enough sachets to cover the period of the study and were asked to return all sachets (used and unused) in order to assess compliance. Participants attended the Wellcome Trust Clinical Research Facility, Southampton General Hospital four times during the study (weeks 0, 4, 6, and 8). At week 4, they received the 2008/2009 seasonal influenza vaccination (Imuvac; Solvay Biologicals, Southampton, UK) by intramuscular injection. In this report, data from the final 4 weeks of the intervention period (i.e., weeks 4, 6, and 8) are presented. Blood and saliva samples were collected at each time point. Preparation of serum, plasma, and peripheral blood mononuclear cells (PBMCs) was described previously (9).

### Assessment of blood immune cell phenotypes and natural killer cell activity

Blood immune cell phenotypes were determined by flow cytometry after staining with relevant fluorescently labeled monoclonal antibody pairs; procedures were performed, as described elsewhere (9). Killing of the K562 (target) cell line by PBMCs was used to assess natural killer (NK) cell activity (9). Killing by unstimulated and interleukin (IL)-2-stimulated PBMCs was determined. Specific target cell lysis was calculated as (% total target cell death − % spontaneous target cell death).

### Measurement of immunoglobulin concentrations in saliva and serum

Salivary secretory IgA (sIgA) and serum total immunoglobulin (Ig)A, IgM, and IgG concentrations were measured by enzyme-linked immunosorbent assay (ELISA) (9).

### Assessment of T-cell responses

Activation of cluster of differentiation (CD)4^+^ T cells was assessed by appearance of CD69 on the cell surface following stimulation for 24 h with the polyclonal T cell mitogen concanavalin A (Con A) at a final concentration of 5 μg/mL (9). The percentage of cells, which had undergone activation (i.e., CD3^+^CD4^+^CD69^+^) as well as the MFI of CD69 expression on these cells, was determined. The concentrations of the cytokines IL-2, IL-4, IL-6, IL-10, tumor necrosis factor (TNF)-α, and interferon (IFN)-γ were measured in the culture medium, as described elsewhere (9). The proliferation of lymphocytes was determined by dilution of carboxyfluorescein diacetate succinimidyl ester (CFSE) over 168 h following Con A stimulation of PBMC cultures, as described elsewhere (9).

### Measurement of serum vaccine-specific antibodies by hemagglutination inhibition assay

Vaccine-specific antibodies in serum were measured by ViroClinics B.V. (Rotterdam, The Netherlands), using a hemagglutination inhibition (HI) assay, based on the principle that influenza viruses agglutinate erythrocytes of some avian species, and incubation of the virus with virus-specific antibodies (present in serum of participants who have been vaccinated) prior to this agglutination reaction will inhibit the agglutination. Antibody titers <10 HI units were set to 5. Seroconversion is defined as the percentage of participants showing at least a fourfold increase in antibody titer and seroprotection as an antibody titer of ≥40 HI units (28).

### Measurement of antibody class-specific, vaccine-specific antibodies by ELISA

Antibody class-specific, vaccine-specific antibodies were measured by a modification of the procedure described by Olivares et al. (29). Ninety-six-well Maxisorb ELISA plates (Fischer Scientific, Loughborough, UK) were coated with a 500 ng/mL solution of vaccine (Solvay Biologicals) in coating buffer (0.5M Na_2_CO_3_ in distilled water), 100 μL/well, and incubated overnight at 4°C. Plates were washed three times with 250 μL wash solution [50 mM TRIS (Aldrich), 0.14M NaCl (Fischer), 1% bovine serum albumin (BSA) (Sigma), 0.2% Tween-20 (Sigma), in distilled water]. One hundred microliters of block buffer [5% BSA in phosphate buffered saline (PBS)] were added to each well and incubated at 37°C for 1 h. Plates were washed three times. Plasma (100 μL undiluted for IgA, IgD, and IgM and diluted 1:100 in PBS for IgG1) was added to each well, and incubated at room temperature for 1 h. Plates were washed three times. Antibody (100 μL mouse anti-human IgA, IgD, IgG1, or IgM; 0.5 mg/mL; AbD Serotec) was added to each well, and incubated at room temperature for 1 h. Plates were washed three times. Goat anti-mouse IgG (H/L):horseradish peroxidize (100 μL; AbD Serotec; diluted 1:10,000 in PBS) was added to each well, and incubated at room temperature for 1 h. Plates were washed three times. Staining was performed by adding 100 μL of 3,3′,5,5′ tetramethylbenzidine (Sigma) and incubating at room temperature in the dark for 20 min. One hundred microliters of stop solution (Sigma) were added, and plates read on a plate reader (Thermo Labsystems, Original Multiskan) at 450 nm. Data are expressed as optical density (OD) units.

### Statistical analysis

Analysis was performed by two-factor ANOVA in the first instance (fixed factors: time and treatment group); data for vaccine-specific antibody titers were log transformed prior to analysis. Where appropriate, comparisons between groups were performed using independent samples *t*-test, Mann–Whitney test, Chi squared test, or Fisher’s exact test depending upon the nature of the data. Comparisons between time points within a group were made using paired *t*-test or Wilcoxon Signed Rank test depending upon the nature of the data. All analyses were performed using SPSS version 17.0 (SPSS Inc., Chicago, IL, USA) and in all cases a value for *P* < 0.05 was taken to indicate statistical significance.

## Results

### Participant characteristics and compliance

Forty-nine participants were recruited and randomized (*n* = 24 in the maltodextrin group; *n* = 25 in the Synergy1 group) and 43 of these completed the study (*n* = 21 in the maltodextrin group; *n* = 22 in the Synergy1 group). The characteristics of these participants, as shown in Table 1, did not differ between the two groups. As reported previously (9) compliance, assessed by returned unused sachets, was good (median 100% in both groups) and Synergy1 increased fecal bifidobacteria numbers.

**Table 1:** **Characteristics according to study group**.

	Maltodextrin group	Synergy1 group
*n*	21	22
Age (years); mean (range)	56 (45–63)	54 (45–62)
Male:female	8:13	3:19
BMI (kg/m^2^); mean (range)	25.0 (17.7–33.8)	25.7 (19.4–33.3)

*Data are shown for participants who completed the study*.

*There were no significant differences between groups*.

### Blood immune cell phenotypes

There were some significant effects of group, but no significant effects of time or significant group × time interactions, for circulating immune cell subsets (Table S1 in Supplementary Material). The percentages of CD3^+^CD8^+^ and CD8^+^ cells were higher in the Synergy1 group (two-factor ANOVA effect of group *P* < 0.001 for both) but were not affected by vaccination. As a consequence of the higher percentage of CD8^+^ cells in the Synergy1 group, this group had a lower CD4:CD8 ratio (two-factor ANOVA effect of group *P* = 0.002).

### Natural killer cell activity

Natural killer cell activity toward K562 cells was enhanced by pre-incubation with IL-2 (Table S2 in Supplementary Material). However, there were no significant effects of group or time and no significant group × time interactions for NK cell activity with or without IL-2 pre-incubation (Table S2 in Supplementary Material).

### Serum total immunoglobulin and salivary IgA concentrations

There were no significant effects of group, one significant effect of time, and no significant group × time interactions for serum total IgA, IgG, and IgM concentrations (Table S3 in Supplementary Material). There was a significant effect of group but no significant effect of time and no significant group × time interaction for salivary sIgA (two-factor ANOVA effect of group *P* = 0.008); concentrations were lower in the Synergy1 group (Table S3 in Supplementary Material). When salivary sIgA concentrations were adjusted for total salivary protein, there was no longer a significant effect of group (Table S3 in Supplementary Material).

### T cell activation, proliferation, and cytokine production in response to polyclonal stimulation

Data for T cell activation, proliferation, and cytokine production, all in response to the polyclonal T cell mitogen Con A, are shown in Table 2. Con A stimulation increased the percentage of CD69 positive CD3^+^CD4^+^ cells and increased the level of CD69 expression on those cells (i.e., MFI) (Table 2). Con A also increased the percentage of proliferating T cells and decreased CFSE MFI indicative of dilution of the dye (Table 2). Finally, Con A increased the production (i.e., the concentration in the culture medium) of all six cytokines assessed (Table 2). Increases were approximately 4-fold for percentage of CD69^+^ cells (from approximately 14 to 55%), approximately 4.5-fold for percentage of proliferating cells (from approximately 15 to 70%), and approximately 2- to 260-fold for cytokine production depending upon the cytokine (see Table 2). There were no significant effects of group, one significant effect of time, and no significant group × time interactions for T cell activation or T cell proliferation (Table 2). There were no significant effects of group or time and no significant group × time interactions for IL-2, IL-6, IL-10, TNF-α, or IFN-γ production in the absence of Con A or IL-2 or IL-10 production in the presence of Con A (Table 2). There was a significant effect of group on production of IL-4 in the absence of Con A (lower in the Synergy1 group, *P* = 0.005) and on production of IL-4 (lower in the Synergy1 group, *P* = 0.032), IL-6 (higher in the Synergy1 group, *P* = 0.032), TNF-α (higher in the Synergy1 group, *P* = 0.024), and IFN-γ (higher in the Synergy1 group, *P* = 0.007) in the presence of Con A. The Th1/Th2 ratio was calculated using the concentrations of the prototypical T helper (Th)1-type (IFN-γ) and Th2-type (IL-4) cytokines. There was a significant effect of group, but not of time and no significant group × time interaction, on the ratio. The ratio was higher in the Synergy1 group (*P* = 0.004).

**Table 2:** **Measures of T cell function in response to Con A in participants in the maltodextrin and Synergy1 groups**.

		Maltodextrin group			Synergy1 group			P*		
		
	ConA	Week 4	Week 6	Week 8	Week 4	Week 6	Week 8	Group	Time	Group × Time
**T Cell Activation**												
% CD3^+^CD4^+^CD69^+^ (%)	−	14.9 (12.6)	12.5 (9.6)	11.5 (8.1)	13.5 (6.2)	15.1 (6.4)	14.9 (9.5)	0.357	0.884	0.439
CD69 MFI	−	45.4 (11.7)	40.1 (10.7)	38.7 (11.5)	45.5 (13.8)	43.3 (10.0)	39.6 (9.8)	0.506	0.049	0.817
% CD3^+^CD4^+^CD69^+^ (%)	+	57.4 (16.1)	55.9 (11.9)	53.3 (16.1)	55.7 (12.6)	52.5 (13.1)	50.6 (14.1)	0.312	0.347	0.964
CD69 MFI	+	101.0 (48.4)	91.9 (33.0)	88.5 (31.7)	92.3 (27.5)	88.4 (25.6)	82.9 (26.5)	0.326	0.335	0.939
**T Cell Proliferation**												
% Proliferating cells (%)	−	17.8 (21.9)	11.6 (5.5)	15.7 (17.9)	16.5 (19.6)	13.1 (14.1)	13.7 (16.3)	0.364	0.774	0.483
CFSE MFI	−	736.8 (316.5)	803.2 (233.2)	661.3 (444.1)	773.1 (292.9)	778.2 (299.9)	815.2 (335.6)	0.862	0.464	0.898
% Proliferating cells (%)	+	66.4 (24.3)	76.6 (15.4)	64.9 (26.6)	69.2 (23.9)	73.2 (20.5)	69.4 (25.1)	0.609	0.516	0.542
CFSE MFI	+	285.4 (326.9)	168.3 (84.9)	258.3 (345.1)	220.2 (163.5)	214.8 (243.1)	208.1 (158.5)	0.769	0.280	0.746
**T cell cytokine production (pg/ml)**												
IL-2	−	2.1 (1.4)	1.9 (0.9)	1.7 (0.7)	1.9 (0.6)	2.1 (1.9)	1.9 (1.1)	0.887	0.629	0.674
IL-4	−	2.1 (1.0)	2.0 (0.9)	1.9 (1.0)	1.6 (0.5)	1.6 (0.4)	1.6 (0.7)	0.005	0.905	0.768
IL-6	−	3036 (3337)	2112 (2275)	2323 (3058)	2639 (3145)	3856 (3415)	3108 (2937)	0.212	0.926	0.299
IL-10	−	16.2 (10.1)	12.9 (9.4)	23.9 (46.9)	18.3 (18.5)	24.7 (23.2)	24.9 (37.3)	0.319	0.471	0.625
TNF-α	−	37.3 (31.0)	42.9 (76.1)	41.7 (43.3)	22.7 (16.8)	60.7 (63.9)	64.9 (75.3)	0.384	0.113	0.257
IFN-γ	−	6.4 (4.4)	4.4 (2.3)	4.1 (1.3)	4.6 (2.3)	11.1 (23.6)	3.7 (0.6)	0.400	0.216	0.117
IL-2	+	97.2 (81.1)	85.7 (81.2)	63.4 (48.7)	109.8 (68.5)	73.7 (55.3)	86.2 (66.1)	0.525	0.125	0.493
IL-4	+	12.2 (9.1)	11.9 (9.7)	10.1 (7.9)	10.8 (15.0)	6.3 (3.8)	6.5 (3.1)	0.032	0.249	0.563
IL-6	+	7235 (5105)	4913 (4058)	4588 (6110)	8310 (3876)	6003 (4371)	8944 (5095)	0.032	0.173	0.304
IL-10	+	76.2 (42.7)	74.2 (66.2)	75.1 (91.8)	92.7 (75.4)	83.1 (119.8)	69.1 (36.1)	0.639	0.764	0.795
TNF- α	+	333.9 (232.6)	270.3 (229.6)	271.5 (261.7)	434.4 (449.1)	388.9 (414.0)	486.5 (439.5)	0.024	0.740	0.730
IFN-γ	+	1056 (1029)	663 (732)	724 (985)	2081 (3127)	1604 (2887)	1754 (2006)	0.007	0.594	0.994
**Th1/Th2 Ratio^a^**												
	−	3.1 (1.7)	2.5 (1.2)	2.4 (0.6)	3.1 (1.4)	6.8 (12.8)	2.5 (0.5)	0.140	0.184	0.135
	+	151.3 (222.4)	102.5 (177.3)	83.4 (97.7)	248.4 (282.4)	181.5 (211.8)	297.3 (369.6)	0.004	0.513	0.397

*Data are mean (SD) for *n* = 21 in the maltodextrin group and *n* = 22 in the Synergy1 group*.

*Value for P from two-factor ANOVA (fixed factors: group, time).

*^a^Calculated using the concentrations of IFN-γ and IL-4*.

*IFN, interferon; IL, interleukin; MFI, median fluorescence intensity; TNF, tumor necrosis factor*.

### Vaccine strain-specific antibody response

Serum vaccine-specific antibody concentrations are shown in Table 3. There was a significant effect of time on the concentrations of all three vaccine-specific antibodies (two-factor ANOVA effect of time *P* < 0.001 for all three antibodies), such that they were higher at weeks 6 and 8 than at week 4. There was a significant effect of group on the concentration of antibodies to the H3N2-like strain (*P* = 0.020), but there was no effect of group on the concentrations of antibodies to the H1N2- or the B-like strains. The antibody response to the H3N2-like strain was greater in the Synergy1 group.

**Table 3:** **Vaccine strain-specific antibody response in participants in the maltodextrin and Synergy1 groups**.

	Maltodextrin group			Synergy1 group			P*		
		
	Week 4	Week 6	Week 8	Week 4	Week 6	Week 8	Group	Time	Group × Time
H1N1	5.3 (1.6)	1103^a^ (3398)	948^a^ (3342)	14.8 (34.3)	518^a^ (1103)	394^a^ (1081)	0.756	<0.001	0.837
H3N2	16.2 (34.1)	4338^a^ (8287)	2702^a^ (6147)	71.9 (277.1)	6234^a^ (8352)	6479^a^ (9035)	0.020	<0.001	0.428
B	6.4 (4.5)	139^a^ (187)	149^a^ (221)	10.7 (17.6)	276^a^ (472)	172^a^ (267)	0.179	<0.001	0.851

*Data are mean (SD) hemagglutination inhibition units for *n* = 21 in the maltodextrin group and *n* = 22 in the Synergy1 group*.

*Value for P from two-factor ANOVA (fixed factors: group, time).

*^a^Significantly different from week 4 (all *P* < 0.001)*.

In the maltodextrin group, seroconversion rates to the H1N1-like, H3N2-like, and B-like strains at week 6 were 85, 70, and 80%, respectively, while in the Synergy1 group, the rates were 85, 90, and 80%, respectively. In the maltodextrin group, seroprotection rates to the H1N1-like, H3N2-like, and B-like strains at week 6 were 80, 72, and 75%, respectively, while in the Synergy1 group, the rates were 80, 91, and 77%, respectively. These rates of serconversion and seroprotection were not significantly different between groups, although they were numerically higher in response to the H3N2-like strain in the Synergy1 group.

### Immunoglobulin class-specific antibodies to the vaccine antigen

Immunoglobulin class-specific antibodies to the vaccine antigen were measured according to the method described by Olivares et al. (29) and, in the absence of standards for these antibodies, data are expressed as OD units (Table 4); OD units are directly related to concentration. There was a significant effect of time on serum vaccine-specific IgM, IgG1, and IgD OD units and a trend to an effect on vaccine-specific IgA OD units (Table 4). In general, these were higher 2 and 4 weeks post-vaccination than prior to vaccination. There was no significant effect of group on vaccine-specific IgA, IgM, or IgD OD units. However, vaccine-specific IgG1 OD units were higher in the Synergy1 group (two-factor ANOVA effect of group *P* = 0.018). At week 6, vaccine-specific IgG1 OD units were higher in the Synergy1 group compared to the maltodextrin group (*P* = 0.028, independent *T*-test). Between week 4 and 6, the increase in specific IgG1 OD units was greater in the Synergy1 group compared to the maltodextrin group (*P* = 0.036, independent *T*-test).

**Table 4:** **Serum vaccine-specific immunoglobulins in participants in the maltodextrin and Synergy1 groups**.

	Maltodextrin group			Synergy1 group			P*		
		
	Week 4	Week 6	Week 8	Week 4	Week 6	Week 8	Group	Time	Group × Time
Specific IgA	1.72 (0.88)	2.20 (1.00)	2.00 (0.97)	1.66 (0.62)	2.03 (0.79)	1.88 (0.82)	0.443	0.076	0.961
Specific IgM	1.75 (0.67)	2.73 (0.73)	2.59 (0.70)	1.85 (0.62)	3.03 (0.63)	2.74 (0.51)	0.133	<0.001	0.759
Specific IgG1	1.18 (0.39)	1.88 (0.57)	2.03 (0.63)	1.26 (0.48)	2.25^a^ (0.48)	2.24 (0.56)	0.018	<0.001	0.441
Specific IgD	0.47 (0.13)	0.58 (0.14)	0.60 (0.14)	0.51 (0.25)	0.58 (0.19)	0.60 (0.22)	0.755	0.012	0.860

*Data are mean (standard deviation) optical density units for *n* = 21 in the maltodextrin group and *n* = 22 in the Synergy1 group*.

*Value for P from two-factor ANOVA (fixed factors: group, time).

*^a^Significantly different from control (maltodextrin) group (*P* = 0.028)*.

## Discussion

Several human studies have investigated the effect of β2-1 fructans upon the antibody response following vaccination, and some of these studies have reported an improvement in this response (16, 17, 19, 20), while others have not (18, 21–26). In these studies, the prebiotic has often been combined with other nutrients (19–26). Furthermore, these previous studies have been conducted in children (16, 18, 21, 23–26) or in elderly adults (17, 19, 20, 22). It is necessary to identify the functional effects β2-1 fructans in the absence of other interventions, and also to assess whether effects occur in middle-aged humans, as this group is considered a target group of consumers. In the current study, a specific combination of long-chain inulin and oligofructose (Synergy1) was evaluated.

The present study investigated the effect of Synergy1 at 8 g/day for 4 weeks prior to, and for 4 weeks following, seasonal influenza vaccination on a range of measures of immune function in healthy middle-aged participants. A randomized controlled parallel study design was used with the digestible carbohydrate maltodextrin as the control. Synergy1 increased fecal bifidobacteria numbers within 4 weeks (9) confirming its prebiotic effect. However, there were no alterations in any of the immune markers measured in the absence of an exogenous immune challenge (i.e., prior to vaccination) (9). It was anticipated that some improvements in immune function would be observed following a controlled immune challenge, such as a seasonal influenza vaccination after supplementation with Synergy1. A range of measures was used in order to identify a potential effect of Synergy1 on immune function. The activity of NK cells was assessed because these cells have a key role in anti-viral immunity and their activity has been shown to be enhanced 2 and 4 weeks following seasonal influenza vaccination (30), although this enhancement was not seen in the current study. Responses of PBMCs to the T cell mitogen Con A were assessed. It is important to note that Con A is a polyclonal T cell stimulant and responses to such stimulants may have only limited relation to the vaccination response. However, an impaired *ex vivo* response to mitogens has been described to occur 2 weeks following seasonal influenza vaccination, with the impairment being associated with a lower vaccine-induced antibody response (31). Again this decline was not seen in the current study. *Ex vivo* PBMC responses to vaccine stimulation were also assessed in the current study but responses were weak and highly variable (data not shown).

Few differences were seen between groups in most immune outcomes measured, including the blood immune cell profile, a marker of mucosal immunity (salivary sIgA), and an innate immune response (NK cell activity). T cell responses to a polyclonal T cell stimulant (Con A) were little affected, although the Th1-type response was higher in the Synergy1 group. However, two important and novel observations were made. First, the antibody response to the H3N2-like strain of the vaccine was higher in the Synergy1 group. Additionally, the seroconversion and seroprotection rates to this strain of the virus tended to be enhanced with Synergy1. Second, the IgG1-specific antibody response to the vaccine (as measured in OD units) was enhanced in the Synergy1 group, and this response appeared to occur more quickly as by week 2 levels had reached a maximum in the Synergy1 group but were still rising in the maltodextrin group. Therefore, Syngery1 was able to enhance some aspects of the antibody response to vaccination, which is considered to be the most valid marker of immune function in humans (13–15).

The enhancement in antibody response to the H3N2-like strain and in vaccine-specific IgG1 suggests that Synergy1 does impact on the host immune system, and this may be the result of the change in fecal (and so gut) microbiotia described previously (9). It is important to identify which aspect of the immune response is affected by Synergy1. The current study focused on identifying whether Synergy1 affected the profile of immune cells in the bloodstream, NK cell activity, and the functional responses of T lymphocytes (activation, proliferation, and cytokine production)-induced *ex vivo* using the polyclonal T cell stimulant Con A. Con A-induced strong activation, proliferation, and cytokine responses. The activation and proliferative responses of T cells were not enhanced by Synergy1, but Con A-induced production of some cytokines was greater with Synergy1, suggestive of an enhanced Th1-type response. This may underlie the enhanced antibody response seen. It is also possible that Synergy1 may have affected antigen presenting cells and the processes of antigen uptake, processing and presentation or B cells and the process of antibody production. These aspects were not investigated here and should be examined in future studies.

The observation that Synergy1 increased the concentration of antibodies to only one of the three strains of the vaccine is consistent with a number of earlier studies with different nutritional and pharmacological interventions. For example, Langkamp-Henken et al. (19) reported that a nutritional formula containing antioxidants, zinc, selenium, β2-1 fructans, and structured triacylglycerol resulted in a higher antibody response to the H1N1-like strain of the influenza virus in elderly subjects, with no effect on the antibody response to the H3N2-like or B-like strains. Boge et al. (32) found that a mix of probiotics resulted in a higher antibody response to the B-like strain at 3, 6, and 9 weeks post-vaccination but with no significant effect on the response to the H1N1-like or the H3N2-like strains. Davidson et al. (33) showed that a mixture of *Lactobacillus casei* GG and β2-1 fructans resulted in a greater response to the H3N2-like stain of the vaccine, with no effect on the response to the H1N1-like or B-like strains. Administration of the steroid hormone dehydroepiandrosterone sulfate resulted in a higher response to the H3N2-like strain of the vaccine in healthy older subjects with no effect on the response to the H1N1-like or B-like strains (34). Furthermore, corticosteroid treatment of asthmatic children and adults impaired the antibody response to the B-like strain but not to the H1N1-like or H3N2-like strains of the vaccine (35). The anti-influenza agent zanamivir resulted in a lower antibody response to the H1N1-like strain of the vaccine in healthy volunteers compared with a placebo treatment, but did not affect the response to the H3N2-like or B-like strains (36). Why these different nutritional and pharmacological interventions consistently influence the response to only one of the three virus strains in the influenza vaccine is not clear, but this may relate to precise nature of the immune interactions between the vaccine antigen and the host immune system and how these interactions are influenced by the intervention.

Strengths of the current study were the use of a randomized, controlled, double-blind design; the high compliance of participants to the intervention (median 100%); the low rate of drop-out (12%); the confirmation that Synergy1 modified the fecal microbiota (9); and the follow-up at two time-points post-vaccination. However, the study does have limitations. First, the relatively small sample size may have limited the ability to identify clear effects of Synergy1. Second, the subjects studied were healthy and middle aged; although they represent a target group for prebiotics the findings cannot be generalized to the elderly or to individuals with disease. Third, as indicated above, we did not examine some aspects of the immune system involved in the response to vaccination.

In conclusion, after supplementation with Synergy1 (8 g/day for 4 weeks prior to vaccination and 4 weeks post-vaccination), there was a higher antibody response to the H3N2-like strain of the vaccine and an enhanced IgG1-specific antibody response to the vaccine. Most other immune responses assessed were not affected, although the Synergy1 group had higher Th1-type responses *ex vivo*. Thus, β2-1 fructans in the form of Synergy1 can enhance some aspects of the immune response in healthy middle-aged adults, but this is not a global effect. However, this effect is relatively modest and its biological significance is not clear.

## Author Contributions

PC designed the research and had overall responsibility for the study; AL recruited the subjects; AL, LC, PN, and EM performed the laboratory analysis; AL, LC, PN, and PC analyzed the data; AL and PC drafted the paper; PC had primary responsibility for its final content. All authors read and approved the final manuscript.

## Conflict of Interest Statement

Philip C. Calder has received consulting fees from Beneo-Orafti. None of the other authors has any conflict to declare.

## Supplementary Material

The Supplementary Material for this article can be found online at http://journal.frontiersin.org/article/10.3389/fimmu.2015.00490

## Abbreviations

BMI, body mass index; BSA, bovine serum albumin; CD, cluster of differentiation; CFSE, carboxyfluorescein diacetate succinimidyl ester; Con A, concanavalin A; ELISA, enzyme-linked immunosorbent assay; HI, hemagglutination inhibition; IFN, interferon; Ig, immunoglobulin; IL, interleukin; MFI, mean fluorescence intensity; NK, natural killer; OD, optical density; PBMC, peripheral blood mononuclear cell; PBS, phosphate buffered saline; sIgA, secretory IgA; Th cell, helper T cell; TNF, tumor necrosis factor.

## References

[1] Roberfroid M Gibson GR Hoyles L McCartney AL Rastall R Rowland I Prebiotic effects: metabolic and health benefits. Br J Nutr (2010) 104(Suppl 2):S1–63. 10.1017/S0007114510003363 20920376

[2] Hill C Guarner F Reid G Gibson GR Merenstein DJ Pot B Expert consensus document: the international scientific association for probiotics and prebiotics consensus statement on the scope and appropriate use of the term probiotic. Nat Rev Gastroenterol Hepatol (2014) 11:506–14. 10.1038/nrgastro.2014.66 24912386

[3] Guigoz Y Rochat F Perruisseau-Carrier G Rochat I Schiffrin EJ Effects of oligosaccharide on the faecal flora and non-specific immune system in elderly people. Nutr Res (2002) 22:13–25. 10.1016/S0271-5317(01)00354-2

[4] Fuller Z Louis P Mihajlovski A Rungapamestry V Ratcliffe B Duncan AJ. Influence of cabbage processing methods and prebiotic manipulation of colonic microflora on glucosinolate breakdown in man. Br J Nutr (2007) 98:364–72. 10.1017/S0007114507709091 17403273

[5] Kolida S Meyer D Gibson GR. A double-blind placebo-controlled study to establish the bifidogenic dose of inulin in healthy humans. Eur J Clin Nutr (2007) 61:1189–95. 10.1038/sj.ejcn.1602636 17268410

[6] Ramnani P Gaudier E Bingham M van Bruggen P Tuohy KM Gibson GR. Prebiotic effect of fruit and vegetable shots containing Jerusalem artichoke inulin: a human intervention study. Br J Nutr (2010) 104:233–40. 10.1017/S000711451000036X 20187995

[7] Kleessen B Schwarz S Boehm A Fuhrmann H Richter A Henle T Jerusalem artichoke and chicory inulin in bakery products affect faecal microbiota of healthy volunteers. Br J Nutr (2007) 98:540–9. 10.1017/S0007114507730751 17445348

[8] Tuohy KM Kolida S Lustenberger AM Gibson GR. The prebiotic effects of biscuits containing partially hydrolysed guar gum and fructo-oligosaccharides – a human volunteer study. Br J Nutr (2001) 86:341–8. 10.1079/BJN2001394 11570986

[9] Lomax AR Cheung LV Tuohy KM Noakes PS Miles EA Calder PC. β2-1 fructans have a bifidogenic effect in healthy middle-aged human subjects but do not alter immune responses examined in the absence of an in vivo immune challenge: results from a randomised controlled trial. Br J Nutr (2012) 108:1818–28. 10.1017/S0007114511007276 22244014

[10] Lomax AR Calder PC. Prebiotics, immune function, infection and inflammation: a review of the evidence. Br J Nutr (2009) 101:633–58. 10.1017/S0007114508055608 18814803

[11] Calder PC Kew S. The immune system: a target for functional foods? Br J Nutr (2002) 88:S165–76. 10.1079/BJN2002682 12495459

[12] Cummings JH Antoine J-M Azpiroz F Bourdet-Sicard R Brandtzaeg P Calder PC PASSCLAIM – gut health and immunity. Eur J Clin Nutr (2004) 43(Suppl 2):II118–73. 10.1007/s00394-004-1205-4 15221356

[13] Albers R Antoine JM Bourdet-Sicard R Calder PC Gleeson M Lesourd B Markers to measure immunomodulation in human nutrition intervention studies. Br J Nutr (2005) 94:452–81. 10.1079/BJN20051469 16176618

[14] Albers R Bourdet-Sicard R Braun D Calder PC Herz U Lambert C Monitoring immune modulation by nutrition in the general population: identifying and substantiating effects on human health. Br J Nutr (2013) 110(Suppl 2):S1–30. 10.1017/S0007114513001505 23902657 PMC3734536

[15] Calder PC. Biomarkers of immunity and inflammation for use in nutrition interventions: International Life Sciences Institute European branch work on selection criteria and interpretation. Endocr Metab Immune Disord Drug Targets (2014) 14:236–44. 10.2174/1871530314666140709091650 25008763

[16] Firmansyah A Pramita G Carrie-Fassler A Haschke F Link-Amster H Improved humoral immune response to measles vaccine in infants receiving infant cereal with fructooligosaccharides. J Pediatr Gastroenterol Nutr (2001) 31:A521.

[17] Bunout D Hirsch S Pia de la Maza M Munoz C Haschke F Steenhout P Effects of prebiotics on the immune response to vaccination in the elderly. JPEN J Parenter Enteral Nutr (2002) 26:372–6. 10.1177/0148607102026006372 12405649

[18] Duggan C Penny ME Hibberd P Gil A Huapaya A Cooper A Oligofructose-supplemented infant cereal: 2 randomized, blinded, community-based trials in Peruvian infants. Am J Clin Nutr (2003) 77:937–42. 12663295 10.1093/ajcn/77.4.937

[19] Langkamp-Henken B Bender BS Gardner EM Herrlinger-Garcia KA Kelley MJ Murasko DM Nutritional formula enhanced immune function and reduced days of symptoms of upper respiratory tract infection in seniors. J Am Geriatr Soc (2004) 52:3–12. 10.1111/j.1532-5415.2004.52003.x 14687308

[20] Langkamp-Henken B Wood SM Herrlinger-Garcia KA Thomas DJ Stechmiller JK Bender BS Nutritional formula improved immune profiles of seniors living in nursing homes. J Am Geriatr Soc (2006) 54:1861–70. 10.1111/j.1532-5415.2006.00982.x 17198491

[21] Moro G Arslanoglu S Stahl B Jelinek J Wahn U Boehm G. A mixture of prebiotic oligosaccharides reduces the incidence of atopic dermatitis during the first six months of age. Arch Dis Child (2006) 91:814–9. 10.1136/adc.2006.098251 16873437 PMC2066015

[22] Bunout D Barrera G Hirsch S Gattas V Pia De la Maza M Haschke F Effects of a nutritional supplement on the immune response and cytokine production in free-living Chilean elderly. JPEN J Parenter Enteral Nutr (2004) 28:348–54. 10.1177/0148607104028005348 15449576

[23] Pérez N Iannicelli JC Girard-Bosch C González S Varea A Disalvo L Effect of probiotic supplementation on immunoglobulins, isoagglutinins and antibody response in children of low socio-economic status. Eur J Nutr (2010) 49:173–9. 10.1007/s00394-009-0063-5 19838618

[24] van den Berg JP1 Westerbeek EA van der Klis FR Berbers GA Lafeber HN van Elburg RM. Neutral and acidic oligosaccharides supplementation does not increase the vaccine antibody response in preterm infants in a randomized clinical trial. PLoS One (2013) 8:e70904. 10.1371/journal.pone.0070904 23951035 PMC3738516

[25] Salvini F Riva E Salvatici E Boehm G Jelinek J Banderali G A specific prebiotic mixture added to starting infant formula has long-lasting bifidogenic effects. J Nutr (2011) 141:1335–9. 10.3945/jn.110.136747 21613452 PMC3113290

[26] Stam J van Stuijvenberg M Garssen J Knipping K Sauer PJ. A mixture of three prebiotics does not affect vaccine specific antibody responses in healthy term infants in the first year of life. Vaccine (2011) 29:7766–72. 10.1016/j.vaccine.2011.07.110 21821078

[27] Available from: http://www.who.int/influenza/vaccines/virus/recommendations/en/

[28] Available from: http://ecdc.europa.eu/en/healthtopics/seasonal_influenza/vaccines/Pages/influenza_vaccination.aspx

[29] Olivares M Diaz-Ropero MP Sierra S Lara-Villoslada F Fonolla J Navas M Oral intake of *Lactobacillus fermentum* CECT5716 enhances the effects of influenza vaccination. Nutrition (2007) 23:254–60. 10.1016/j.nut.2007.01.004 17352961

[30] Schapiro JM Segev Y Rannon L Alkan M Rager-Zisman B. Natural killer (NK) cell response after vaccination of volunteers with killed influenza vaccine. J Med Virol (1990) 30:196–200. 10.1002/jmv.1890300310 2341835

[31] Huang YP Pechere JC Michel M Gauthey L Loreto M Curran JA In vivo T cell activation, in vitro defective IL-2 secretion, and response to influenza vaccination in elderly women. J Immunol (1992) 148:715–22. 1730868

[32] Boge T Rémigy M Vaudaine S Tanguy J Bourdet-Sicard R van der Werf S A probiotic fermented dairy drink improves antibody response to influenza vaccination in the elderly in two randomised controlled trials. Vaccine (2009) 27:5677–84. 19615959 10.1016/j.vaccine.2009.06.094

[33] Davidson LE Fiorino AM Snydman DR Hibberd PL. Lactobacillus GG as an immune adjuvant for live-attenuated influenza vaccine in healthy adults: a randomized double-blind placebo-controlled trial. Eur J Clin Nutr (2011) 65:501–7. 10.1038/ejcn.2010.289 21285968 PMC3071884

[34] Degelau J Guay D Hallgren H. The effect of DHEAS on influenza vaccination in aging adults. J Am Geriatr Soc (1997) 45:747–51. 10.1111/j.1532-5415.1997.tb01482.x 9180672

[35] Hanania NA Sockrider M Castro M Holbrook JT Tonascia J Wise R Immune response to influenza vaccination in children and adults with asthma: effect of corticosteroid therapy. J Allergy Clin Immunol (2004) 113:717–24. 10.1016/j.jaci.2003.12.584 15100679

[36] Cox RJ Mykkeltvedt E Sjursen H Haaheim LR. The effect of zanamivir treatment on the early immune response to influenza vaccination. Vaccine (2001) 19:4743–9. 10.1016/S0264-410X(01)00219-5 11535325

