# Supplementary material for: Inulin-Type β2-1 Fructans have Some Effect on the Antibody Response to Seasonal Influenza Vaccination in Healthy Middle-Aged Humans
Source: Front Immunol. 2015 Sep 22;6:490. doi: 10.3389/fimmu.2015.00490 (PMC4585271; doi:10.3389/fimmu.2015.00490)
Supplement: Supplementary file 1 [file Table_1.PDF]

Supplemental TABLE 1. Immune cell phenotypes in whole blood in participants in the maltodextrin and Synergy1 groups

	Maltodextrin group			Synergy1 group			P*		
Cell phenotype	Week 4	Week 6	Week 8	Week 4	Week 6	Week 8	Group	Time	Group x Time
$CD3^{+}CD4^{+}$ (% of lymphocytes)	50.5 (6.9)	51.8 (7.5)	51.7 (5.9)	50.1 (8.4)	50.7 (8.4)	51.2 (7.6)	0.610	0.766	0.971
$CD4^{+}$ (% of lymphocytes)	50.9 (7.1)	52.2 (7.6)	51.8 (5.9)	50.3 (8.3)	50.9 (8.3)	51.4 (7.6)	0.567	0.808	0.972
$CD3^{+}CD8^{+}$ (% of lymphocytes)	17.3 (7.1)	17.2 (6.6)	17.8 (6.9)	22.9 (8.9)	22.7 (9.0)	23.2 (9.4)	< 0.001	0.953	0.999
$CD8^{+}$ (% of lymphocytes)	21.0 (7.4)	20.9 (7.3)	21.4 (7.2)	26.7 (8.0)	26.7 (8.1)	27.1 (8.5)	< 0.001	0.967	1.000
$CD3^{-}CD16^{+}$ (% of lymphocytes)	9.4 (3.7)	10.1 (4.4)	9.8 (5.1)	8.2 (5.5)	8.1 (5.9)	8.5 (5.9)	0.114	0.946	0.923
$CD3^{-}CD19^{+}$ (% of lymphocytes)	10.8 (3.7)	10.6 (3.4)	9.9 (3.3)	10.0 (3.9)	9.8 (3.8)	9.1 (3.8)	0.247	0.484	0.999
$CD14^{+}$ (% of monocytes)	77.9 (13.8)	78.3 (16.9)	84.3 (9.8)	76.9 (14.3)	81.7 (11.9)	81.9 (11.9)	0.991	0.152	0.590
$CD14^{+}$ (% of leukocytes)	3.6 (1.5)	3.6 (1.0)	3.8 (1.2)	3.2 (0.6)	3.5 (1.1)	3.4 (0.9)	0.158	0.842	0.783
$CD127^{lo}$ (% of $CD4^{+}CD25^{+}$ cells)	73.4 (8.3)	71.9 (11.4)	68.4 (12.3)	73.1 (8.6)	71.7 (8.1)	71.5 (9.4)	0.634	0.304	0.654
$CD4^{+}CD25^{+}CD127^{lo}$ (% of total leukocytes)	1.2 (0.6)	1.1 (0.4)	1.3 (0.5)	1.2 (0.5)	1.2 (0.5)	1.1 (0.4)	0.388	0.850	0.446
CD4:CD8	2.8 (1.1)	2.8 (1.3)	2.7 (1.0)	2.1 (0.9)	2.2 (0.9)	2.2 (0.9)	0.002	0.907	0.926

Data are mean (standard deviation) for n = 21 in the maltodextrin group and n = 22 in the Synergy1 group.

*Value for P from ANOVA (fixed factors: group, time)
