# Supplementary material for: Inulin-Type β2-1 Fructans have Some Effect on the Antibody Response to Seasonal Influenza Vaccination in Healthy Middle-Aged Humans
Source: Front Immunol. 2015 Sep 22;6:490. doi: 10.3389/fimmu.2015.00490 (PMC4585271; doi:10.3389/fimmu.2015.00490)
Supplement: Supplementary file 2 [file Table_2.PDF]

Supplemental TABLE 2. Natural killer cell activity (% specific target cell lysis) in participants in the maltodextrin and Synergy1 groups

		Maltodextrin group			Synergy1 group			*P*[*]		
		Week 4	Week 6	Week 8	Week 4	Week 6	Week 8	Group	Time	Group x Time
- IL-2	E:T 100:1	19.0 (8.5)	16.5 (6.6)	18.2 (6.5)	12.4 (5.6)	14.5 (5.9)	17.1 (8.4)	0.093	0.575	0.468
- IL-2	E:T 50:1	12.5 (6.7)	10.7 (4.6)	13.1 (5.4)	9.6 (4.2)	11.3 (4.6)	11.7 (5.6)	0.317	0.563	0.520
- IL-2	E:T 25:1	7.4 (4.4)	6.6 (3.2)	9.7 (5.1)	8.4 (5.4)	9.5 (4.0)	8.8 (4.5)	0.328	0.515	0.298
- IL-2	E:T 12.5:1	5.9 (3.6)	5.4 (2.6)	7.3 (3.7)	5.2 (3.2)	6.2 (3.8)	6.0 (3.8)	0.629	0.488	0.499
+ IL-2	E:T 100:1	37.0 (14.0)	29.7 (11.4)	30.5 (9.1)	25.5 (13.7)	30.4 (14.9)	26.7 (15.7)	0.219	0.858	0.426
+ IL-2	E:T 50:1	26.6 (12.3)	22.9 (10.6)	27.7 (9.6)	19.0 (14.0)	20.9 (11.6)	20.1 (13.4)	0.112	0.901	0.750
+ IL-2	E:T 25:1	14.5 (7.3)	14.6 (8.5)	15.8 (7.5)	18.0 (17.6)	14.1 (8.8)	16.4 (13.3)	0.697	0.837	0.870
+ IL-2	E:T 12.5:1	9.9 (6.0)	8.3 (4.2)	11.0 (7.0)	7.2 (5.9)	6.6 (3.1)	8.0 (8.8)	0.157	0.611	0.946

Data are mean (standard deviation) for n = 21 in the maltodextrin group and n = 22 in the Synergy1 group.

[*]Value for P from ANOVA (fixed factors: group, time).

E:T, effector to target cell ratio; IL, interleukin.
