# Supplementary material for: Inulin-Type β2-1 Fructans have Some Effect on the Antibody Response to Seasonal Influenza Vaccination in Healthy Middle-Aged Humans
Source: Front Immunol. 2015 Sep 22;6:490. doi: 10.3389/fimmu.2015.00490 (PMC4585271; doi:10.3389/fimmu.2015.00490)
Supplement: Supplementary file 3 [file Table_3.PDF]

Supplemental TABLE 3. Total serum immunoglobulin and salivary immunoglobulin A concentrations in participants in the maltodextrin and Synergy1 groups

| | Maltodextrin group | | | Synergy1 group | | | *P*[*] | | |
|---|---|---|---|---|---|---|---|---|---|
| | Week 4 | Week 6 | Week 8 | Week 4 | Week 6 | Week 8 | Group | Time | Group x Time |
| IgA (mg/mL) | 2.5 (1.6) | 2.8 (1.3) | 2.5 (1.3) | 2.6 (1.6) | 3.4 (0.9) | 2.6 (1.5) | 0.244 | 0.105 | 0.768 |
| IgG (mg/mL) | 30.3 (16.6) | 20.0 (10.0) | 18.6 (13.4) | 36.1 (38.4) | 16.2 (6.7) | 27.8 (28.3) | 0.770 | 0.121 | 0.620 |
| IgM (mg/mL) | 0.9 (0.4) | 0.9 (0.4) | 0.7 (0.3) | 0.8 (0.4) | 0.9 (0.4) | 0.8 (0.5) | 0.343 | 0.007 | 0.370 |
| sIgA/total protein (µg/mg) | 261.1 (192.1) | 348.9 (436.0) | 355.7 (256.6) | 340.5 (391.4) | 344.0 (280.5) | 343.1 (343.0) | 0.726 | 0.750 | 0.776 |
| sIgA (µg/mL) | 233.8 (100.9) | 227.2 (105.5) | 221.4 (100.4) | 205.5 (94.1) | 162.8 (81.1) | 176.5 (87.6) | 0.008 | 0.439 | 0.682 |

Data are mean (standard deviation) for n = 21 in the maltodextrin group and n = 22 in the Synergy1 group.
[*]Value for P from ANOVA (fixed factors: group, time)
